# Survival outcomes of patients with germ cell tumors treated with high-dose chemotherapy for refractory or relapsing disease

**DOI:** 10.18632/oncotarget.25162

**Published:** 2018-04-27

**Authors:** Stefanie Zschäbitz, Florian A Distler, Benjamin Krieger, Patrick Wuchter, Kerstin Schäfer-Eckart, Maximilian Jenzer, Markus Hohenfellner, Peter Dreger, Georg Martin Haag, Dirk Jäger, Sascha Pahernik, Carsten Grüllich

**Affiliations:** ^1^ Department of Medical Oncology, National Center for Tumor Diseases, University Hospital Heidelberg, 69120 Heidelberg, Germany; ^2^ Department of Urology, Paracelsus Medical University, 90419 Nuremberg, Germany; ^3^ Department of Oncology and Hematology, Paracelsus Medical University, 90419 Nuremberg, Germany; ^4^ Department of Hematology, Oncology, and Rheumatology, University Hospital Heidelberg, 69120 Heidelberg, Germany; ^5^ Department of Urology, University Hospital Heidelberg, 69120 Heidelberg, Germany; ^6^ Present address: Institute for Transfusion Medicine and Immunology Mannheim, Medical Faculty Mannheim, University of Heidelberg, DRK-Blutspendedienst Baden-Württemberg–Hessen gGmbH, 68167 Mannheim, Germany

**Keywords:** autologous stem cell transplantation, high-dose chemotherapy, germ cell tumor, relapse, testicular cancer

## Abstract

**Introduction:**

Male patients with metastatic germ cell tumors can be cured in up to 96% of cases depending on stage and IGCCCG prognosis group. Treatment in relapse consists of conventional or high-dose chemotherapy (HDCT) with autologous stem cell transplantation (ASCT) combined with local treatment modalities.

**Results:**

Most patients were classified as poor risk according to IGCCCG (*n* = 24; 52%) and as intermediate (*n* = 12), high (*n* = 16), or very high risk (*n* = 9) at time of first relapse according to IPFSG criteria. In 67% of patients (*n* = 31) HDCT/ASCT was performed as first salvage treatment in relapse or for primary refractory disease following first line chemotherapy. In 46% of patients (*n* = 21) progressive disease was documented after mobilization and prior to HDCT/ASCT. Median progression free survival (mPFS) was 7.4 months (95% confidence interval (CI): 1.3–13.6) while median overall survival (mOS) was 22.2 months (95% CI: 8.9–35.5). When stratified for IPFSG risk group, mPFS (*p* < 0.001) and mOS (*p* = 0.009) differed significantly between risk groups (very low vs. low vs. intermediate vs. high vs. very high). Metastases to liver/bone/brain and platinum refractory disease were independent risk factors for inferior PFS (*p* = 0.024; *p* = 0.008) but not OS.

**Materials and Methods:**

Forty-six patients treated with HDCT/ASCT at the university clinics in Heidelberg and Nuremberg between 2000–2016 were identified and analyzed. Data was collected retrospectively.

**Conclusions:**

HDCT/ASCT offers a potential curative strategy for patients with relapsed GCT. Improvement is still needed in patients with intermediate, high, and very high IPFSG risk group.

## INTRODUCTION

Germ cell tumors (GCT) are the most common type of cancer in men between the ages of 15 and 35. Even in first and potentially second relapse metastatic GCTs can be treated in curative intent. Depending on a set of clinical parameters at the time of diagnosis (known as International Germ Cell Cancer Collaborative Group (IGCCCG) score) patients can be classified into three different risk groups (good, intermediate, and poor) [1]. According to stage and IGCCCG risk group first line platinum-based treatment is scheduled. In case of relapse, the International Prognostic Factors Study Group (IPFSG) prognosis score can be calculated considering primary site and histology, response to prior treatment, progression-free interval, tumor markers in salvage situation, as well as presence of liver, bone, or brain (LBB) metastases. Five prognostic groups result (very low, low, intermediate, high, and very high risk) indicating the further course of disease [2]. Survival rates have significantly improved by the 1990s due to platinum containing chemotherapy regimens and advanced surgical techniques. Relapse within four weeks after platinum containing treatment is considered to be platinum refractory disease [3]. High-dose chemotherapy (HDCT) with subsequent autologous stem cell transplantation (ASCT) is besides conventional dose chemotherapy (CDCT) a potential second salvage treatment that can achieve a 5 year survival rate of about 20% in multiple relapsed GCTs [4]. Single and sequential HDCT/ASCTs approaches have been described. Most centers use high dose carboplatin and etoposide in a sequential mode as this has been associated with a more favourable toxicity profile in a prospective phase 3 trial [5].

Whether HDCT and ASCT are the optimal salvage treatment strategy in first relapse is an unsolved question that is currently investigated with an international randomized phase III trial – the TIGER trial (ClinicalTrials.gov number, NCT02375204) [6]. Within this study CDCT (i.e. paclitaxel, ifosfamide, and cisplatin (TIP)) is compared with HDCT using paclitaxel for mobilizing stem cells and high dose carboplatin and etoposide (TI-CE) – one of the most commonly used HDCT regimens.

A current meta-analysis of 59 trials found a trend for better median OS (mOS) in HDCT patients analysing data from 1781 and 2447 patients respectively [7]. However, results were not statistical significant at 1, 2, and 5 years of follow-up. Pooled treatment related mortality rates with CDCT and HDCT were 1.29 and 6.46%, respectively (*p <* 0.001).

Typical acute toxicities of HDCT/ASCT – besides hematological side effects – include gastrointestinal, hepatic, infectious, pulmonary and renal complications. Most commonly noted fatal complications of HDCT/ASCT are sepsis and hepatic failure [8].

This manuscript presents registry data from two German university medical centers regarding treatment outcomes of male patients with metastasized GCTs post HDCT and ASCT.

## RESULTS

### Patient characteristics

We identified 46 patients with metastatic GCT that underwent HDCT/ASCT between 2000–2016 (Table 1). Thirty-nine patients were treated in Heidelberg and *n* = 7 patients in Nuremberg. Age range was 15 – 57 years (median: 33 years). All patients had at least an acceptable performance status (ECOG ≤ 2) and all had adequate end-organ function. Most patients had mixed type tumor (*n* = 24, 52%). Six patients (13%) had pure seminoma. Thirteen (28%), *n* = 21 (46%) and *n* = 25 (54%) patients had metastases to liver, bone and/or brain at time of diagnosis, first relapse and of HDCT/ASCT, respectively.

**Table 1:** Patient characteristics at initial diagnosis (*n* = 46)

Characteristic	No.	%
Median age, years	33	
Range, years	15–57	
Primary tumor site		
Gonadal	35	76
Mediastinal	9	20
Extragonadal	2	4
Histologic type at initial diagnosis		
Mixed tumors	24	52
Seminoma	6	13
Yolk sac	3	7
Embryonal	6	13
Chorion	4	9
Teratocarcinoma	1	2
Unknown	2	4
IGCCCG risk group at initial diagnosis		
Good	15	33
Intermediate	4	9
Poor	24	52
Unknown	3	7
IPFSG risk group at first relapse		
Very low	3	7
Low	6	13
Intermediate	12	26
High	16	35
Very high	9	20

Abbreviations: IGCCCG: International Germ Cell Cancer Collaborative Group; IPFSG: International Prognostic Factors Study Group.

Most patients were classified as poor risk group according to IGCCCG (*n* = 24; 52%; good risk: *n* = 15; 33%; “intermediate risk” *n* = 4; 9%; unknown: *n* = 3; 7%) and as high risk according to IPFSG (*n* = 16; 35%; intermediate: *n* = 12; 26%; very high: *n* = 9; 20%; low: *n* = 6; 13%; very low: *n* = 3; 7%) at time of first relapse. This distribution was unchanged when IPFSG was calculated with characteristics of relapse at time of HDCT/ASCT. However, one patient was upgraded from low to intermediate IPFSG score due to higher tumor marker levels in second relapse when he underwent HDCT/ASCT while another patient was downgraded from intermediate to low risk IPFSG score (also due to tumor marker levels). In *n* = 10 patients (22%) IPFSG score sum had changed from first relapse to relapse prior to HDCT/ASCT with no consequence to IPFSG risk category.

Indication for HDCT/ASCT was relapse in *n* = 35 (76%) patients, primary refractory state to first line treatment in *n* = 8 (17%) patient, and the approach to consolidate the result of CDCT in *n* = 1 patient (2%) with unfavorable characteristics. In *n* = 2 patients (4%) HDCT/ASCT was performed to consolidate the results of local treatment. Of these, in one patient with mixed type NSGCT and mature teratoma component experiencing a second relapse thoracic surgery was performed to receive information on the histology of the tumor. R0 status could be achieved and after pathological evaluation revealed non-teratomatous malignant components, HDCT/ASCT was performed. The other patient presented with a singular cerebral filia in first relapse and received stereotactical radiotherapy in curative dose before proceeding to HDCT/ASCT.

In 67% of patients (*n* = 31) HDCT/ASCT was performed as first salvage regimen. Fifteen patients (33%) underwent HDCT/ASCT in higher relapse (*n* = 12 in second relapse, *n* = 2 in third relapse, *n* = 1 in fourth relapse). 17 patients (37%) were platinum refractory at the time of HDCT/ASCT.

In 46% (*n* = 21) of patients progressive disease (PD) was documented following mobilization chemotherapy and prior to first HDCT/ASCT; 7 patients were in stable disease (15%) and *n* = 16 (33%) in partial remission (PR) or complete remission (CR) after mobilization chemotherapy or local treatment.

### Transplantation characteristics and transplant related toxicities

In 12 patients single (*n* = 8, 17%) or tandem transplantation (*n* = 4, 9%) was planned and performed. However, for most patients (*n* = 34, 74%) a sequential approach with three cycles was chosen. In *n* = 25 patients all three cycles of HDCT/ASCT were applied. In *n* = 6 patients less than the initially anticipated number of cycles of HDCT/ASCT was applied due to treatment toxicity or death: Two patients suffered from neutropenic sepsis or fever in combination with mucositis CTC IV° and their performance status deteriorated dramatically without a soon recovery which made further HDCT/ASCT impossible. Another patient developed sepsis without a clinical focus with complications IV° (acute renal failure, ST segment elevation myocardial infarction requiring stent implantation after first HDCT cycle). In another patient progressive neuropathy III° led to discontinuation of HDCT. One patient died due to a septic course of pneumonia, another patient due to neutropenic colitis with toxic megacolon. In *n* = 3 patients (7%) progressive disease was noted prior to second or third HDCT. Therefore further cycles of HDCT/ASCT were omitted and a palliative treatment approach was hence initiated.

### Consolidating treatment after HDCT/ASCT

Further consolidation therapy after HDCT/ASCT was as follows: In *n* = 19 patients (41%) resection of residual tumor was performed. In *n* = 1 patient (2%) surgery was planned but interrupted at exploration due to unresectable disease. One patient (2%) in whom surgical resection of a medistinal residual tumor was technically impossible received radiotherapy in curative intention. In *n* = 2 patients (4%) definitive local treatment was performed prior to HDCT/ASCT as stated above.

In the remaining 23 patients (50%) no residual tumor resection or radiotherapy was performed subsequently to HDCT/ASCT. Reasons were: uncontrolled progressive disease (*n* = 9, 20%), complete remission (*n* = 6, 13%), death (*n* = 3, 7%), markedly reduced performance score (Karnofsky index 30% or lower, *n* = 2, 4%), decline per patient (*n* = 2, 4%), or reason not documented (*n* = 1, 2%).

In patients with residual tumor resection vital tumor was found in *n* = 13 samples (68%), among those were *n* = 2 patients in whom residual mature teratoma only was present. In *n* = 3 samples (16%) tumor was avital and in *n* = 3 (16%) viability of tumor cells was not reported.

Characteristics of the subgroup of patients who were in CR post HDCT/ASCT with or without local treatment are given in Supplementary Table 1.

### Progression free and overall survival

**Figure 1:**
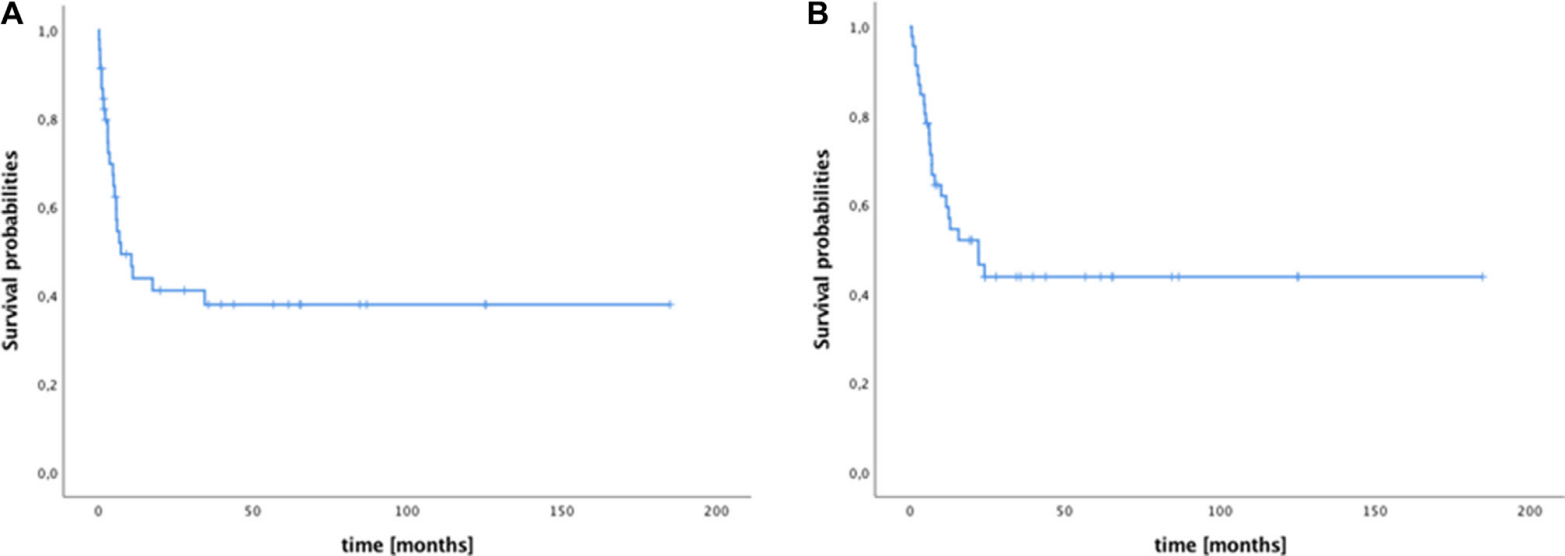
Survival of patients with metastatic germ cell tumors after high-dose chemotherapy/autologous stell cell transplantation (**A**) Median progression free survival (mPFS) after high-dose chemotherapy (HDCT)/autologous stem cell transplantation (ASCT); median PFS was 7.4 months. (**B**) Median overall survival (mOS) after HDCT/ASCT was 22.2 months.

Median progression free survival (PFS). of all patient was 7.4 months (95% confidence interval (CI): 1.3–13.6, Figure 1A) while median OS was 22.2 months (95% CI: 8.9–35.5, Figure 1B). Six patients are alive and in remission at a follow up < 24 months while *n* = 16 patients are alive and in remission at > 24 months.

**Figure 2:**
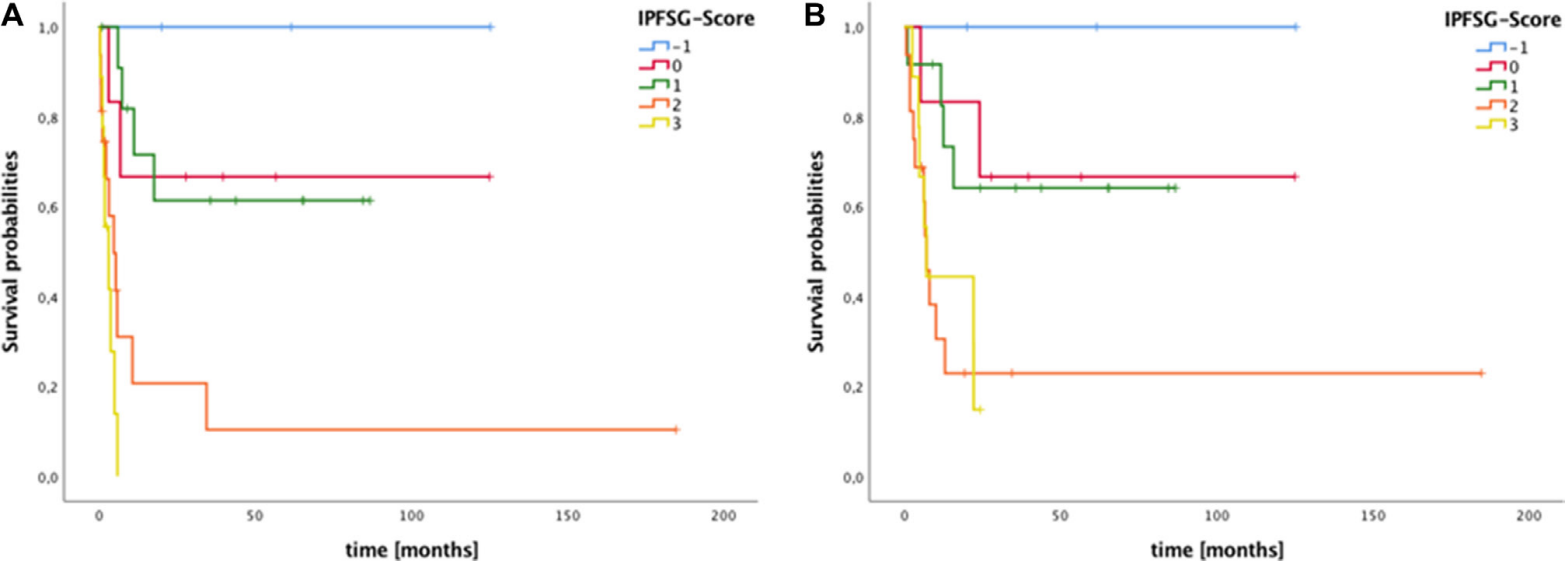
Survival of patients with metastatic germ cell tumors stratified by International Prognostic Factors Study Group (IPFSG) score (**A**) Median progression free survival (mPFS) after high-dose chemotherapy (HDCT)/autologous stem cell transplantation (ASCT) stratified by IPFSG score differed significantly between risk groups (log rank *p <* 0.001). (**B**) Median overall survival (mOS) after HDCT/ASCT stratified by IPFSG also score differed significantly (log rank *p* = 0.009).

When stratified for IPFSG group mPFS and mOS differed significantly between risk groups (*p <* 0.001, and *p* = 0.009, Figure 2A and 2B). Median PFS and OS were not reached in the very low, low, and intermediate risk groups. Median PFS was 4.8 and 3.1 months for the high and very high risk group (95% CI: 1.3–8.6 and 0.0–6.4), mOS was 7.2 and 7.2 months (95% CI: 5.1–9.2 and 4.9–9.5).

**Figure 3:**
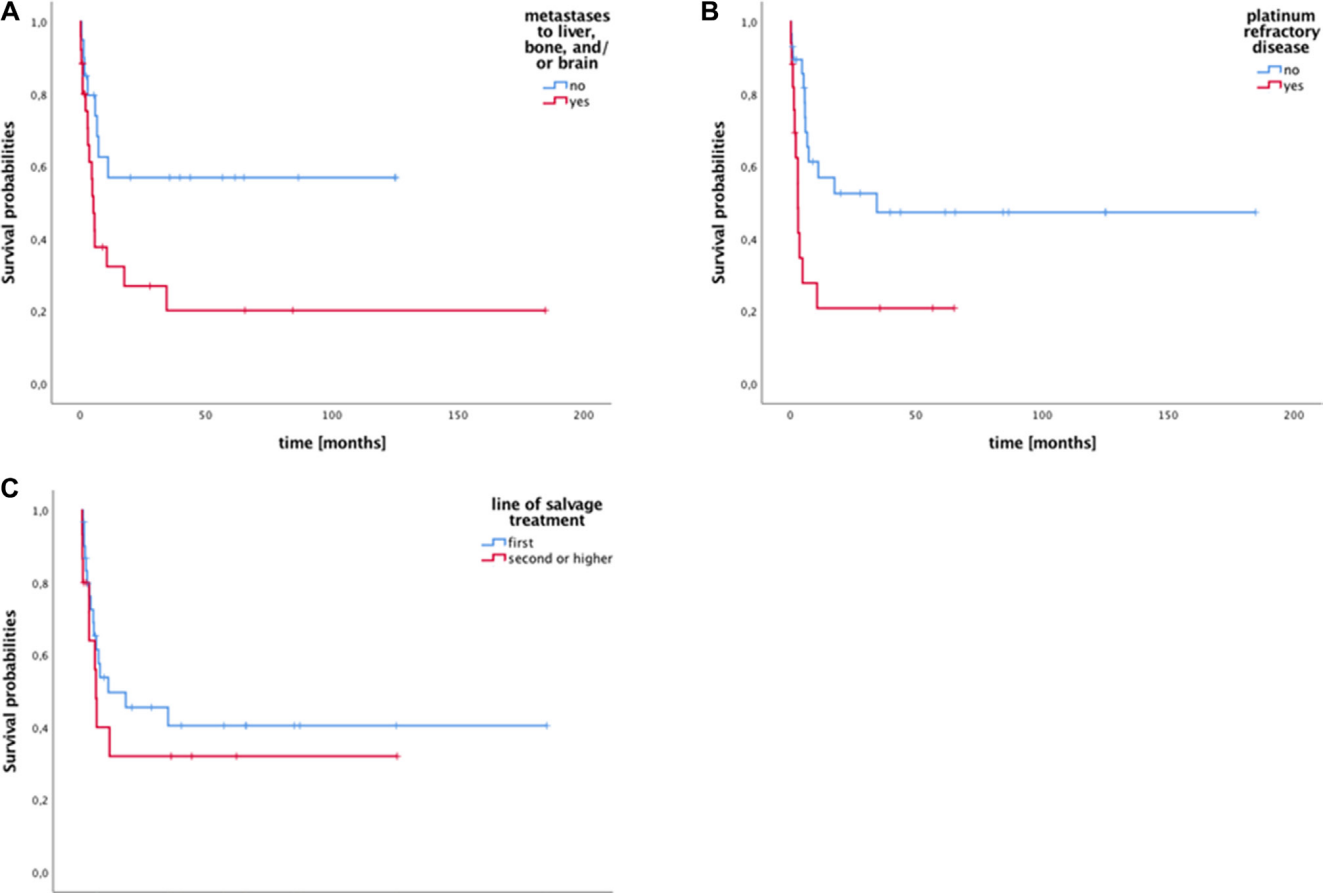
Survival of patients with metastatic germ cell tumors stratified by existence of metastases to liver, brain and/or bone (LBB), platinum refractory disease, and transplantation in first vs. further relapse (**A**) Median progression free survival (mPFS) after high-dose chemotherapy (HDCT)/autologous stem cell transplantation (ASCT) stratified by LBB metastases differed significantly (log rank *p* = 0.011). (**B**) Median PFS after HDCT/ASCT differed significantly between patients with platinum responsive or refractory disease at time of HDCT/ASCT (log rank *p* = 0.008). (**C**) Transplantation upfront or in first relapse vs. second or higher relapse was neither significant for mPFS (10.8 months vs. 5.9 months; *p* = 0.41 Figure 3C) or mOS (not reached vs. 13.0 months; *p* = 0.21).

Stratification for existence of LBB metastases and platinum responsive/refractory disease at time of HDCT/ASCT was associated with significant differences in mPFS for both factors. Median PFS for patients with no LBB metastases was not reached vs. 5.4 months when LBB metastases were present (95% CI: 3.8–7.0; *p* = 0.024; Figure 3A). For platinum responsive vs. unresponsive disease mPFS was 34.5 months vs. 3.2 months (95% CI: 1.3–13.6; *p* = 0.008), respectively, Figure 3B). However, both factors were not associated with significant differences in mOS. For patients without LBB metastases mOS was not reached compared to the LBB metastasis group with mOS of 10.2 months (95% CI: 1.2–19.2; *p* = 0.085). For platinum responsive patients mOS was not reached vs. 6.7 months (95% CI: 4.2–9.2; *p* = 0.076) for the platinum refractory cohort.

Between transplantation for primary refractory disease or in first relapse compared to second or higher relapse no significant differences for mPFS or mOS were detected. Median PFS from HDCT/ASCT for the primary refractory/first relapse group was 10.8 months (95% CI: 0.0–41.1 months) compared to 5.9 months (95% CI: 4.8–7.1 months) for the second or consecutive relapse group (*p* = 0.41; Figure 3C). Median OS from HDCT/ASCT for patients who received HDCT/ASCT as first salvage treatment vs. in second and later relapse was not reached vs. 13.0 months (95% CI: 8.9–35.5; *p* = 0.21).

**Figure 4:**
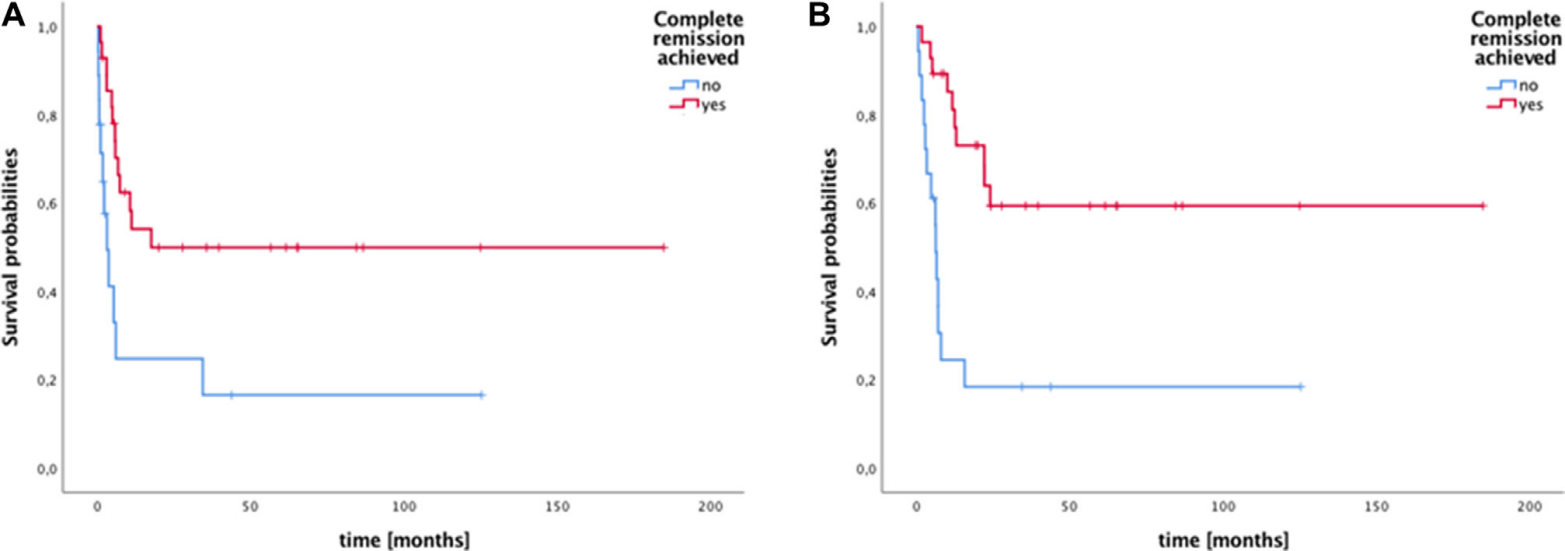
Survival of patients with metastatic germ cell tumors stratified by achievement of complete remission (CR) to high-dose chemotherapy (HDCT)/autologous stell cell transplantation (ASCT) +/- local treatment (**A**) Median progression free survival (mPFS) in patients with CR was not reached and was 3.3 months when no CR was achieved (*p <* 0.01). (**B**) Median overall survival (mOS) in patients with CR was not reached vs. 13.0 months when no CR was achieved (*p <* 0.01).

In patients who were in complete remission after HDCT/ASCT and those who received residual tumor resection or radiotherapy as consolidation mPFS was 17.7 months (range 2–185; 95% CI: n.a.) and mOS has not been reached with 64% of patients being alive at a median follow up time of 41 months (Figure 4A and 4B). Median PFS and OS in patients in whom no CR was achieved or no additive local treatment was performed was 3.3 months (95% CI: 1.0–5.5) and 6.4 months (95% CI: 5.6–7.2), respectively. Both patients who declined residual tumor resection as well as the patient where the information is missing on why no local additive treatment was performed are alive with no signs of progression at follow-up times of 34, 43, and 125 months, respectively, indicating that residual lesions (which were present on imaging) were rather fibrosis or necrosis than viable tumor.

Of all patients with an OS of less than 3 months, 5 out of 6 patients were treated prior to 2010. Causes of death were toxicity of HDCT/ASCT (*n* = 2), progressive disease (*n* = 3) and fatal complications of residual tumor resection (*n* = 1).

## DISCUSSION

Metastatic GCTs are a rare though potentially curative disease. While a major proportion of patients will be cured with first line treatment, a subset of patients requires salvage treatment for relapsing or primary refractory disease. The ideal sequence of treatment beyond first line has not been established to date. The international TIGER trial is actively recruiting patients aiming to solve the question of superiority of CDCT or HDCT as first relapse regimen. This study evaluates the outcomes of 46 patients with metastatic germ cell tumors who underwent HDCT/ASCT in different treatment lines and were treated at two German university medical centers between 2000 and 2016.

It is widely accepted that patients with relapsed metastatic GCT should be referred to centers of testicular cancer excellence for further treatment planning. Improved outcomes after hematopoietic stem cell transplantation have been repeatedly described in high volume transplant centers compared to low- or middle-volume centers– however, the threshold for what is high-volume has not been clearly defined [9, 10]. In our cohort treatment-related mortality was 4.3%, which is above what has been reported from the Indiana Center recently (i.e. 2.4%) but in line with the experience of other centers [8, 11]. However, in our cohort the proportion of patients proceeding to HDCT/ASCT in 2nd relapse rather than first relapse was much higher than in the Indiana cohort mentioned above (33% vs. 19%). Higher toxicity levels (including CTC grade 5 toxicity) have been described previously in patients with more intensive pretreatment caused for example by more severe myelosuppression possibly explaining the phenomenon [12]. Also, in our cohort the proportion of patients with dismal prognostic features was higher (e.g. IPFSG score distribution very low – low – intermediate – high – very high: 6.5 – 13.0 – 26.1 – 34.8 – 19.6% (Heidelberg/Nuremberg) *versus* 11 – 18 – 25.5 – 25.5 – 20% (Indiana) or tumor site: mediastinal primary 18% (Heidelberg/Nuremberg) vs. 5% (Indiana)).

When stratified for IPFSG risk groups PFS and OS differed significantly between groups as previously described in larger cohorts [4, 8]. The IPFSG score has been validated for first relapse only. However, we calculated it for the fifteen patients who underwent HDCT/ASCT in second or higher relapse with characteristics present at HDCT/ASCT. The IPFSG score had changed in two of the patients upgrading one from low to intermediate risk and downgrading the other patient vice versa. We abstained from calculating survival curves for patients who underwent HDCT/ASCT in second or higher relapse stratified to IPFSG score prior to HDCT/ASCT due to the small sample size. However, It would be interesting to retrospectively or prospectively analyze the prognostic value of IPFSG score calculated in second relapse.

While others could demonstrate a PFS/OS advantage for platinum sensitive disease, in our cohort only PFS but not OS was improved. However, the analysis of the subgroup of patients who achieved a CR after HDCT or after definitive local treatment post HDCT/ASCT reveals a relevant proportion of patients with long term survival albeit dismal characteristics such as presence of LBB metastases (*n* = 14, 50.0%), platinum refractory disease (*n* = 9, 32.1%), mediastinal primary (*n* = 6, 21.4%), as well as a very high or high IPFSG risk score (*n* = 11, 39.3%). This emphasizes the importance of multimodal and interdisciplinary treatment planning for those patients.

In our cohort, HDCT/ASCT in first vs. second or higher salvage situation did not show a significant difference in mOS and mPS. However, the latter subgroup was relatively small and meaningful statements cannot be derived. In most high volume centers the decision regarding CDCT or HDCT in first relapse will be made considering the individual patients’ risk factors. Results from the TIGER trial are urgently awaited for better guidance.

Limitations of our data are its retrospective acquisition, heterogenous treatment regimens, and small sample size. Due to the retrospective approach and documentation we were not able to identify patients in whom HDCT/ASCT might have been a rational treatment approach but was abandoned due to factors such as low performance status and co-morbidities. Owing to the small subgroups we were not able to conclude efficacy data on different treatment regimens. Toxicity data, however, speak in favor for a sequential approach with high dose carboplatin end etoposide an can to date be regarded as standard regimen. In cases where stem cell yield allows for one transplant only, single ASCT and a cyclophosphamide or thiotepa containing single HDCT regimen may still play a role. In addition, for patients with CNS involvement (brain metastases, leptomeningeal disease), the good penetration of thiotepa into the CNS might be a rationale for chosing a single HD regimen containing thiotepa.

Unfortunately, there is still a subset of patients with dismal prognostic characteristics not responding to or relapsing after HDCT/ASCT. Palliative chemotherapy with gemcitabine, oxaliplatin, and paclitaxel (GOP) might offer disease control in combination with local procedures in a small proportion of these patients [13, 14]. However, in the majority of patients GOP stabilizes the disease for a short time only. Several drug classes have been or currently are investigated in patients for whom no standard treatment options exist as summarized in a recent review [15] among them sunitinib, cabazitaxel, brentuximab and checkpoint inhibitors. None of these substances has been proven overwhelmingly successful although in single patients positive courses of treatment have been described. This is leaving a strong clinical need for further research regarding strategies for molecular diagnostics and innovative therapeutics.

## MATERIALS AND METHODS

### Patients

We retrospectively analyzed all patients with metastatic GCTs treated with HDCT/ASCT at the university medical centers in Heidelberg and Nuremberg between 2000 and 2016. Medical information on the clinical courses including survival was retrieved from the prospective electronical patient charts [16], from the local tumor registries, involved primary care physicians and medical oncologists.

Clinical parameters assessed included tumor stage, histology, sites of metastases, type of mobilization chemotherapy, type of HDCT, number of HDCT/ASCT cycles, types of prior and subsequent treatment regimens, tumor marker and imaging responses as well as data on toxicity (grade, type of side effects). The IPFSG score was determined as previously described [2]. It was calculated with characteristics at first relapse of disease and in case of HDCT/ASCT in further relapse with characteristics prior to mobilization chemotherapy.

Primary endpoint was OS, secondary endpoint was PFS post HDCT/ASCT.

Peripheral-blood stem cells were harvested after bone marrow stimulation with chemotherapy followed by application of granulocyte colony-stimulating factor (GCSF). In cases of unsuccessful stem cell harvest mobilization chemotherapy was changed to another regimen and/or plerixafor, a CXCR4-inhibitor, was used additionally. Chemotherapy (mobilizing, HDCT) was administered ad described in Supplementary Table 2.

CR was defined as the disappearance of all tumor manifestations on radiographic studies and normalization of tumor markers. PR was defined as reduction in tumor size on imaging with completely (PRm−) or incompletely (PRm+) normalized tumor markers. PD was defined as an increase in tumor size on imaging or rising tumor markers. Stable disease was defined as response that did not meet criteria for CR, PR or PD.

The project was approved by the local ethics committee (EKHD 0115).

### Statistical analysis

PFS and OS were calculated from the date of first transplantation to the date of relapse and death or last follow-up, respectively. Survival and progression were calculated using Kaplan-Meier estimates and compared using log-rank tests. *P*-values less than 0.05 were considered statistically significant. Statistical analyses were conducted using the SPSS v25 software.

## CONCLUSIONS

In conclusion, HDCT/ASCT offers a curative treatment approach in relapsed and even platinum-refractory metastatic germ cell cancer patients especially when a CR can be achieved. Whether to use HDCT in first or second relapse is a matter of debate. A subset of relapsed patients does not benefit from HDCT/ASCT. New treatment strategies are urgently needed for this patient population.

## SUPPLEMENTARY MATERIALS TABLES



## Abbreviations

ASCT: autologous stem cell transplantation
CDCT: conventional dose chemotherapy
CE: carboplatin etoposide
CEC: carboplatin etoposide cyclophosphamide
CEI: carboplatin etoposid ifosfamide
CET: carboplatin etoposide thiotepa
CIT: carboplatin ifosfamide paclitaxel
CTC: common toxicity criteria
ET: etoposide thiotepa
GCSF: granulocyte colony-stimulating factor
GCT: germ cell tumor
HDCT: high-dose chemotherapy
IGCCCG: International Germ Cell Cancer Collaborative Group
IPFSG: International Prognostic Factors Study Group
LBB: metastases to liver, brain and/or bone
(m)OS: (median) overall survival
PEI: cisplatin etoposide ifosfamide
(m) PFS: (median) progression free survival
TI: paclitaxel ifosfamide
TIP: paclitaxel ifosfamide cisplatin
