# Supplementary material for: Survival outcomes of patients with germ cell tumors treated with high-dose chemotherapy for refractory or relapsing disease
Source: Oncotarget. 2018 Apr 27;9(32):22537–45. doi: 10.18632/oncotarget.25162 (PMC5976483; doi:10.18632/oncotarget.25162)
Supplement: Supplementary file 1 [file oncotarget-09-22537-s001.pdf]

Survival outcomes of patients with germ cell tumors treated with high-dose chemotherapy for refractory or relapsing disease

SUPPLEMENTARY MATERIALS

Supplementary Table 1: Patient characteristics of subgroup with complete remission or additive local treatment post HDCT/ ASCT (*n* = 28)

Characteristic	No.	%
Median age, years	36	
Range, years	15–59	
Primary tumor site		
Gonadal	22	79
Mediastinal	6	21
Extragonadal	0	0
IPFSG risk group at first relapse		
Very low	2	7
Low	6	21
Intermediate	9	32
High	6	21
Very high	5	18
LBB metastases at HDCT/ASCT		
Yes	14	50
No	14	50
Platinum responsive disease		
Yes	19	68
No	9	32
Salvage line of HSCT/ASCT		
First	22	79
Second	6	21
≥ Third	0	0

Abbreviations: ASCT: autologous stem cell transplantation; HDCT: high dose chemotherapy; IGCCCG: International Germ Cell Cancer Collaborative Group; IPFSG: International Prognostic Factors Study Group.

Supplementary Table 2: Mobilization chemotherapy and high dose chemotherapy regimen

Mobilization chemotherapy	High dose chemotherapy (HDCT)	Number of HDCT cycles conducted/ anticipated	Reason for treatment discontinuation	Number of patients	Annotation
CEI	CE	3/3		2	
CEI	other (CE-CE-ET)	3/3		1	Change of HDCT type due to complications
PEI	CE	3/3		13	
		2/3	PD	1	
		2/3	complications	2	
		1/3	complications	2	
		2/2		2	
		1/1		2	
PEI	CEC	2/2		1	
		1/1		2	
PEI	CET	1/1		1	
PEI	PEI	3/3		2	
		2/2		1	
PEI	PEI-Paclitaxel	3/3		1	
		2/3	PD	1	
PEI	other	3/3		1	PEI-Paclitaxel --> PD --> 3 cycles CE
TI	CE	3/3		1	
TI	PEI-Paclitaxel	3/3		1	
TI	other (PEI-Paclitaxel-PEI-CEI	3/3		1	Change of HDCT type due to complications
TIP	CE	3/3		2	
		2/3	complications	1	
		1/1		1	
TIP	CET	1/1		1	
TIP	other (PEI-CIT)	2/3	PD	1	Change of HDCT type due to PD
None (plerixafor + GCSF)	CET	1/1		1	
None (GCSF only)	CE	1/3	complications	1	

Abbreviations: CE: carboplatin etoposide; CEC: carboplatin etoposide cyclophosphamide; CEI: carboplatin etoposid ifosfamide; CET: carboplatin etoposide thiotepa; CIT: carboplatin ifosfamide paclitaxel; ET: etoposide thiotepa; HDCT: high dose chemotherapy; PEI: cisplatin etoposide ifosfamide; PD: progressive disease; TI: paclitaxel ifosfamide; TIP: paclitaxel ifosfamide cisplatin.
